# Preparation of Polyaniline-Modified Cellulose/PDMS Composite Triboelectric Material and Application of Its Pretreatment in MOW Pulp

**DOI:** 10.3390/polym16101413

**Published:** 2024-05-16

**Authors:** Xiaoping Sun, Yuhe Wei, Yanfen Sun, Juan Yuan, Haoqiu Chen, Zhuo Chen, Mengyang Wang, Lianxin Luo

**Affiliations:** Guangxi Key Laboratory of Clean Pulp & Papermaking and Pollution Control, School of Light Industry and Food Engineering, Guangxi University, Nanning 530004, China; 2116301044@st.gxu.edu.cn (X.S.); weiyuhegxu@outlook.com (Y.W.); sunyanfen1128@163.com (Y.S.); 13786777813@163.com (J.Y.); 2216301001@st.gxu.edu.cn (H.C.); 2316391004@st.gxu.edu.cn (Z.C.); mengyang1616@163.com (M.W.)

**Keywords:** cellulose, PDMS, polyaniline, TENG, deinking

## Abstract

Self-powered electronic equipment has rapidly developed in the fields of sensing, motion monitoring, and energy collection, posing a greater challenge to triboelectric materials. Triboelectric materials need to enhance their electrical conductivity and mechanical strength to address the increasing demand for stability and to mitigate unpredictable physical damage. In this study, polyaniline-modified cellulose was prepared by means of in situ polymerization and compounded with polydimethylsiloxane, resulting in a triboelectric material with enhanced strength and conductivity. The material was fabricated into a tubular triboelectric nanogenerator (TENG) (G-TENG), and an electrocatalytic pretreatment of mixed office waste paper (MOW) pulp was performed using papermaking white water as the flowing liquid to improve the deinking performance. The electrical output performance of G-TENG is highest at a flow rate of 400 mL/min, producing a voltage of 22.76 V and a current of 1.024 μA. Moreover, the deinking effect of MOW was enhanced after the electrical pretreatment. This study explores the potential application of G-TENG as a self-powered sensor power supply and emphasizes its prospect as an energy collection device.

## 1. Introduction

With energy shortages and environmental issues becoming increasingly prominent, research on low-carbon, clean, and sustainable energy technologies has garnered significant attention [1,2,3,4]. In 2012, the team of Wang et al., led by academician Wang, first proposed an efficient energy collection device, the triboelectric nanogenerator (TENG), based on the principle of contact electrification and electrostatic induction coupling [5,6,7]. This device can convert low-frequency irregular mechanical energy into electrical energy. TENG can collect wind energy, wave energy, solar energy, heat energy, and more, offering unique advantages such as its lightweight design, low cost, simple manufacturing process, and high power density [7,8]. In recent years, TENG research has gradually extended to numerous fields. Several studies have explored the use of TENG to collect energy from liquid flows and apply the resulting electricity in research areas such as catalytic degradation [9,10].

Many triboelectric materials, known for their excellent properties, are nonrenewable synthetic polymers. With the development of sustainable industrial practices and the demand for new materials, advocating the use of green and environmentally friendly materials has become the mainstream of current research. Hence, there is an urgent need to find green and low-cost materials to replace nondegradable polymer materials. Polydimethylsiloxane (PDMS), which is used in this study, demonstrates great potential for TENG applications because of its nontoxicity, good flexibility, transparency, biocompatibility, simple preparation, and low cost [11,12,13,14].

However, owing to issues such as weak conductivity and low mechanical properties, many green materials seriously compromise the stability and accuracy of triboelectricity, thus limiting their potential for application in TENG [15]. Researchers have made numerous attempts to enhance the electrical properties of materials by introducing functional groups [16], increasing the dielectric constant [17], and designing micropatterned surfaces [18]. The imine groups in polyaniline (PANI) serve as suitable electron-releasing groups, thereby improving the triboelectric properties of PDMS [19]. Currently, network structures designed through hydrogen bonding [20], supramolecular recognition [21], complexation, electrostatic interaction [22], hydrophobic association [23], and other noncovalent interactions have been developed and applied to strengthen triboelectricity. Therefore, adding fibers can create a uniform network structure, thereby enhancing the mechanical strength of PDMS composites.

Papermaking white water is a suspension composed of charged particles including fibers, fine fibers, filler particles, and polyelectrolytes [24,25]. In addition to these common charged particles, the composition of waste paper white water is more complex, including deinking chemicals, residual ink, and secondary adhesives (DCS) [26,27]. These substances cause problems such as mesh blockage, shortened mesh service life, reduced paper whiteness, and inferior physical properties during the recycling process of waste paper. Under applied potential, charged particles undergo electrolysis, increasing particle interaction and altering concentration and existing forms. These changes may affect the physical state and chemical properties of the solution.

In this study, PANI-modified cellulose was prepared by means of in situ polymerization of aniline on cellulose. The modified cellulose was then compounded with PDMS to improve the electrical output performance and mechanical strength of the PDMS material. Subsequently, the composite PDMS material was made into a tubular solid–liquid TENG, which was used to collect energy generated by contact between liquids and solids to generate electricity. The electrocatalytic pretreatment of mixed office waste paper (MOW) pulp was performed using papermaking white water as the flowing liquid to improve deinking performance. G-TENG achieved an output voltage of 22.76 V, a current of 1.024 μA, and a charge of 8.252 nC, resulting in reduced ink residue in the waste paper pulp and improved pulp whiteness. This study serves as a reference for the collection of the liquid flow energy and the application of converted electric energy in the production process.

## 2. Materials and Methods

### 2.1. Materials

Microcrystalline cellulose and ammonium persulfate (APS) were obtained from Shanghai Aladdin Biochemical Technology Co., Ltd in Shanghai, China. Aniline, hydrochloric acid (HCl), NaOH, H_2_O_2_, NaSiO_3_, EDTA, and OP10 were purchased from Tianjin Damao Chemical Reagent Factory in Tianjin, China. Polydimethylsiloxane (PDMS) was sourced from Dow Corning Company, Midland, MI, USA. Whereas cellulase, xylanase, amylase, and lipase were purchased from Hunan Haizheng Biotechnology Co., Ltd in Jinshi City, China.

### 2.2. Preparation of PANI-Modified Cellulose

PANI-modified cellulose was prepared by means of chemical oxidative polymerization at 0 °C with aniline as the monomer, cellulose as the site stabilizer, 1 M HCl as the dopant, and ammonium persulfate (APS) as the initiator. And the materials were dried through freeze-drying. The dosage ratio of lignocellulose, aniline, and APS was 1:1:1.25.

### 2.3. Preparation of PDMS Composites

The dried PANI-modified cellulose was dispersed in a PDMS solution using ultrasonic waves for 0.5 h. Subsequently, PDMS was cured using TEOS as the curing agent. The ratio of PDMS to Tetraethyl Orthosilicate (TEOS) was maintained at 100:10. After vacuum defoaming, the resulting liquid was poured into a mold to create the film material, which was then cured at 60 °C for 1 h.

### 2.4. Preparation of Tubular TENG

The PDMS composite material was poured into a special mold, and the outer layer was covered with a copper tube to create a tubular material with an outer diameter of 10 mm, an inner diameter of 6 mm, and a length of 40 mm. The tubular material was then connected using a silicone tube.

### 2.5. TENG’s Handling of MOW

MOW was crushed and filtered through a 200-mesh filter screen using a dynamic water filter (DDJ) to obtain white water. DCS was obtained by centrifugation at 2000 rpm for 20 min. G-TENG treated 8% pulp for 30 min, followed by chemical and enzymatic treatments.

### 2.6. Measurement and Characterization

Various analytical techniques were used, including XRD (MINFLEX600, Rigaku Corporation, Akishima, Japan), FTIR (IRTracer-100, Shimadzu Corporation, Kyoto, Japan), SEM (Sigma 300, Carl Zeiss AG, Jena, Germany), nanoparticle size and potential analysis (NANO ZS90, Malvern, UK), XPS (ESCALAB 250XI), thermogravimetric analysis (STA449F3), AFM (Bruker Dimension ICON), conductivity analysis (ST2643), and broadband dielectric impedance analysis (Agilent 4294A). The contact output of PACM-TENG was controlled by a linear motor (LinMot E1100, Spreitenbach, Switzerland). An electrometer (Keithley 6514, Portland, OR, USA) and a data acquisition card (NI-USB6259, Austin, TX, USA) were used to test the output current, voltage, and transfer charge of TENG under the conditions of 20 kPa and 1.5 Hz at room temperature. A pressure sensor (Hychuan, Bengbu, China) was used to control the contact output pressure of the linear motor.

## 3. Results and Discussion

### 3.1. Design Principle of PANI-Modified Cellulose/PDMS Triboelectric Material and Tubular TENG

**Figure 1:**
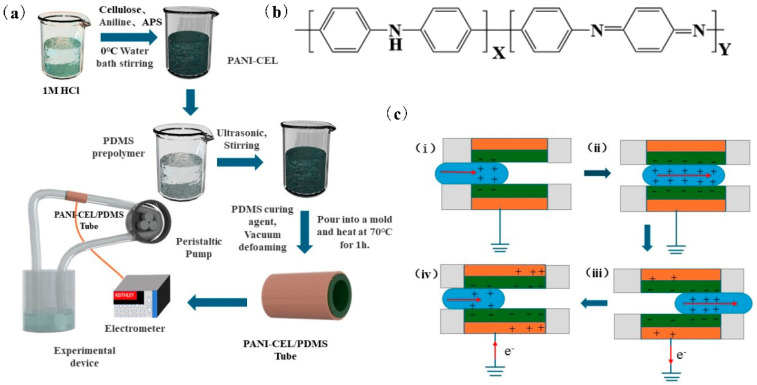
Design principle of PANI-modified cellulose/PDMS triboelectric material and tubular TENG. (**a**) Preparation process and experimental device of PANI-modified cellulose/PDMS composite pipeline material. (**b**) Schematic diagram of PANI molecular structure. (**c**) Solid–liquid contact friction within pipeline initiating mechanism. (**i**) Liquid begins to enter the pipeline. (**ii**) The liquid flows in the pipeline. (**iii**) The liquid leaves the pipeline. (**iv**) New liquid enters the pipeline.

Figure 1a shows a schematic diagram of the preparation process and the experimental device used to prepare the PANI-modified cellulose/PDMS composite pipeline material. PANI-modified cellulose was prepared by means of in situ polymerization, and it was compounded with PDMS to make tubular materials in the mold.

The sample was connected to a silicone tube, and a peristaltic pump was used to transport liquid to collect electrical signals. PANI consists of two basic structural units, namely, “benzene–benzene” and “benzene–quinone” (Figure 1b). Under the influence of protonic acid, its imine group forms a bipolaron, which then transitions into a polaron [28,29]. Polarons and bipolarons exhibit delocalization, contributing to the conductivity of PANI. Figure 1c shows the working principle of the pipeline TENG: (i) As uncharged liquid enters the pipeline, according to the electric double layer theory, the surface of PDMS will be negatively charged, whereas the interface between the liquid and the solid becomes positively charged. (ii) The electrification process of the liquid continues as it flows through the pipeline flow. A large number of negative charges accumulate on the surface of the PDMS material at the contact surface with the liquid until reaching equilibrium and maintaining this state for a duration of time. (iii) As the liquid continues to flow out of the pipeline, there is no more charge on the surface of PDMS for induction, resulting in the formation of a negative potential difference between the copper electrode and the ground. Electrons are then transferred from the copper electrode to the ground, generating an instantaneous negative current. (iv) When fresh liquid enters the pipeline, because the surface of PDMS has a negative charge and there is no more charge for induction, a new positive potential difference is formed between the copper electrode and the ground. Electrons flow from the ground to the copper electrode, creating a positive current. This cycle of liquid outflow and entry repeats thereafter [30].

### 3.2. Structural Characteristics of Composite Materials

#### 3.2.1. PANI Surface Morphology and Structural Characterization of Cellulose before and after PANI Modification

**Figure 2:**
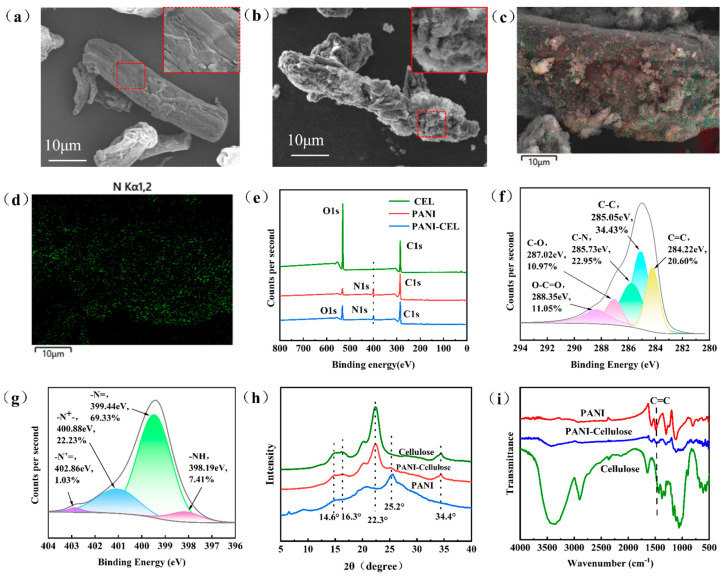
Surface morphology and structure characterization of PANI and cellulose before and after modification. (**a**) SEM image of cellulose surface. (**b**) SEM image of surface of PANI-modified cellulose surface. (**c**) Surface element distribution diagram of PANI-modified cellulose. (**d**) Distribution diagram of nitrogen in PANI-modified cellulose. (**e**) XPS full spectrum. (**f**) C1s spectrogram. (**g**) N1s spectrogram. (**h**) X-ray diffraction pattern. (**i**) Infrared spectrogram.

The surface morphology and structural characteristics of cellulose before and after modification are shown in Figure 2. The surface of cellulose appeared relatively clean, with a few branches, as shown in Figure 2a. Following modification, numerous substances with rough surfaces were observed to adhere to the cellulose surface (Figure 2b), indicating that PANI was well wrapped onto the cellulose surface during polymerization. The distribution of elements modified by PANI (Figure 2c,d) reveals that a large number of uniformly dispersed nitrogen elements appeared on the surface of the modified cellulose. After modification, the binding energy peak of N1s appeared (Figure 2e), and the binding energy peaks of C–N and C=C appeared at 285.73 and 284.22 eV in C1s, respectively (Figure 2f), which were not observed in cellulose. The N1s spectrum (Figure 2g) reveals the presence of binding energy peaks at 402.86 and 400.88 eV, corresponding, respectively, to –N^+^= and –N^+^– bonds, which are unique bonds not present in the raw material aniline [31,32]. It is demonstrated that aniline underwent oxidation and polymerization into PANI on cellulose, becoming attached to the surface of cellulose, which imparts good electrical conductivity to the composites. Figure 2i shows that the modified cellulose retained the diffraction peaks of cellulose itself at 16.2°, 22.3°, and 34.3°, corresponding to the crystal planes of cubic silver crystals (111), (200), and (004), respectively. However, the diffraction peaks belonging to PANI appeared near 25°, corresponding to the (200) crystal plane. This indicates that PANI was aligned along the (200) crystal plane. The modified cellulose retained the characteristic absorption peak of cellulose (Figure 2i), and the C=C stretching vibration absorption peaks of the aniline quinone ring and benzene ring appeared, confirming that PANI was self-polymerized on cellulose [33,34,35].

#### 3.2.2. Characterization of Surface Morphology and Structure of PDMS Composites

**Figure 3:**
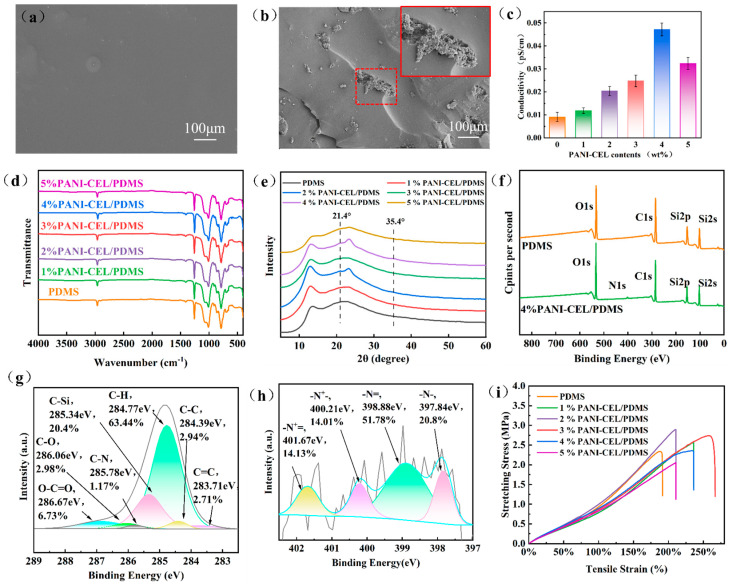
Surface morphology and structural characterization of PDMS composites. (**a**) SEM image of surface. (**b**) SEM image of section. (**c**) Electrical conductivity of composite material. (**d**) Infrared spectrum. (**e**) XRD pattern. (**f**) XPS full spectrum. (**g**) C1s spectrogram. (**h**) N1s spectrogram. (**i**) Tensile properties.

The surface of the composite prepared by the reaction of modified PANI and PDMS was smooth (Figure 3a), but the presence of PANI-modified cellulose can be clearly observed in the cross section of the composite (Figure 3b), tightly wrapped within the middle layer by PDMS. The conductivity of the composites is shown (Figure 3c); with the increase in PANI-modified cellulose content, the conductivity of the PDMS composites was improved, and the maximum conductivity of the samples with 4% content was 4.72 × 10^−2^ pS/cm, which was beneficial to the improvement in the electrical output performance. Infrared spectrum analysis reveals that all samples have similar curve peaks (Figure 3d). Because of the tight packing of PDMS and the small amount of PANI-modified cellulose, it is not possible to observe the obvious absorption peaks of cellulose and PANI. XRD analysis (Figure 3e) reveals that the composite PDMS exhibits only slight fluctuations at about 21.4° and 35.4°, which correspond to the (111) and (004) diffraction peaks of cellulose, respectively. The full spectrum of Figure 3f reveals the appearance of the characteristic peak of N elements after adding the modified PANI cellulose. The C1s spectrum (Figure 3g) exhibits a few binding energy peaks belonging to PANI cellulose, whereas the N1s binding energy (Figure 3h) displays a binding energy peak similar to that of PANI-modified cellulose [36,37,38], in which –N^+^= and –N^+^– help the composite to obtain higher conductivity. In the tensile stress–strain test of the samples (Figure 3i), the stress of the samples with PANI-modified cellulose was improved. Because the fibers can form a uniform network structure, the samples of the 3% PANI-modified cellulose/PDMS composite achieved a maximum strain of 273.82%.

### 3.3. Triboelectric Properties of PDMS Composites

**Figure 4:**
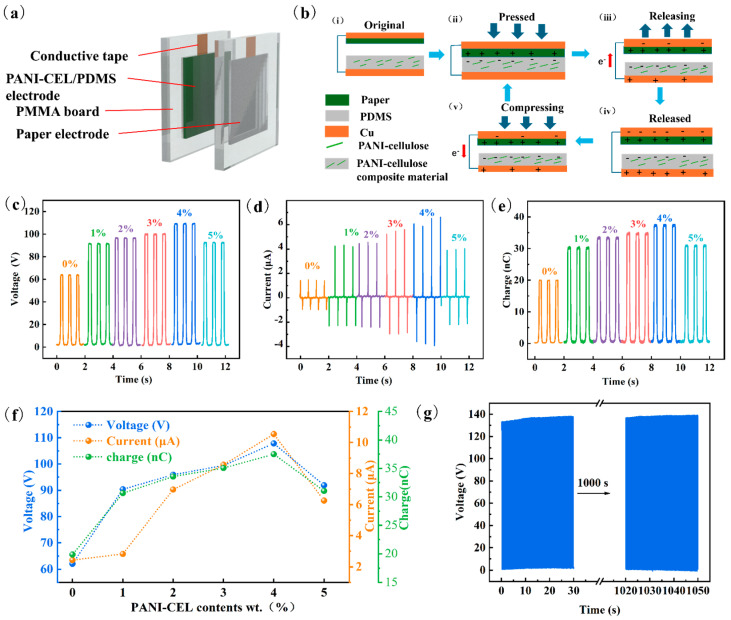
Electrical output performance of PDMS composite in TENG device. (**a**) Structural diagram. (**b**) Process and schematic diagram of contact output. (**i**) original state, (**ii**) pressed state, (**iii**) releasing state, (**iv**) released state, and (**v**) compressing process state. (**c**) Voltage. (**d**) Current. (**e**) Charge density. (**f**) Changing trend of TENG’s electrical output performance. (**g**) Output voltage of TENG working for 1000 s.

Owing to the excellent mechanical properties and electrical conductivity of PDMS composites, they were used as electrodes in the TENG devices. Figure 4a shows the structural schematic illustration. Figure 4b shows the working principle of TENG, in which periodic contact between triboelectric materials drives electrons to flow back and forth in an external circuit. The composite material with PANI-modified cellulose exhibits a higher output performance (Figure 4c–f), attributed to the imine group in PANI increasing the conductivity. Specifically, the electrical output performance of the composite with 4% PANI-modified cellulose content is optimal, with a voltage of 107.82 V, a current of 10.54 μA, and a charge is 37.49 nC, which represent increases of 174%, 434%, and 189%, respectively. Stability and durability (1000 s) tests were conducted to confirm the stability of the TENG electrical output produced by the composite PDMS (Figure 4g).

### 3.4. PDMS Composite Pipeline Electrical Output

**Figure 5:**
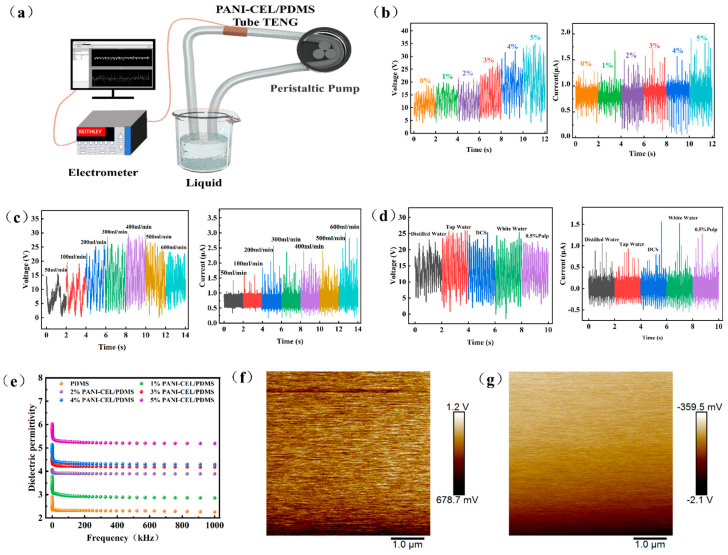
Electrical output performance of PDMS composite in G-TENG device for liquid transportation. (**a**) Schematic illustration of G-TENG device. (**b**) Output voltage and current with varying PANI-modified cellulose content. (**c**) Output voltage and current with different flow rates. (**d**) Output voltage and current in different liquids. (**e**) Short-circuit current and dielectric properties of PANI-modified cellulose with different contents. (**f**) Surface potential of pure PDMS. (**g**) Surface potential of composite PDMS.

Based on the excellent mechanical properties and electrical output performance of the PDMS composite material, it was prepared into a pipeline TENG for collecting liquid and generating electricity through contact with the composite material during the flowing process. A schematic illustration of the device is shown in Figure 5a. With the increase in PANI-modified cellulose content, G-TENG with tap water exhibited a better electrical output performance (Figure 5b). This is because the conductivity of the PDMS composites increased with the addition of PANI-modified cellulose, and the output voltage and current were highest when the PANI-modified cellulose content was 5%, resulting in increases of 227% and 184% in the voltage and current, respectively. With the increase in liquid flow rate, the velocity of liquid contact with the pipeline accelerated, becoming relatively sufficient, and the output voltage and current of G-TENG gradually increased until reaching the peak at approximately 400 mL/min (Figure 5c). As the flow rate continued to increase, there was not enough induced charge between the liquid and the pipeline, leading to a drop in the voltage and current. The electrical output performance of G-TENG with different liquids varied (Figure 5d). It can be observed that it is more beneficial to improve the electrical output performance of G-TENG with more soluble substances in the liquid. The dielectric properties of triboelectric materials significantly affect the electrical output performance of TENG. PDMS with modified cellulose exhibited a higher dielectric constant (DK > 2.5) (Figure 5e), indicating a greater ability to store charge. As shown in Figure 5f,g, the surface potential of PDMS decreased from 678.7 mV to 1.2 V, then to −2.1 V, and finally to −359.5 mV. After the addition of PANI-modified cellulose, the surface potential further decreased, indicating higher electronegativity.

### 3.5. Application of G-TENG Self-Powered Pretreatment in MOW Pulp

**Figure 6:**
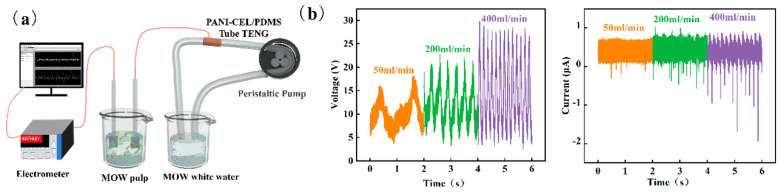
G-TENG self-powered pretreatment of MOW pulp. (**a**) Schematic illustration of device for treating MOW with G-TENG. (**b**) Output voltage and current in MOW white water G-TENG at different flow rates.

Figure 6a shows a schematic illustration of the device for G-TENG electro-pretreatment of MOW pulp. The flowing liquid was MOW white water, and the raw material used for electro-pretreatment was 8% MOW pulp. In the process of white water flowing, the triboelectricity generated by the white water and its contact with the pipe wall was collected, and the electrical output performance of G-TENG increased with the increase in liquid flow rate (Figure 6a). When the white water and the PDMS tube made contact, some induced charges accumulated on the particles in the white water. The increasing charges altered the force between suspended particles, which impacted the substances in the white water and changed the charge characteristics of the papermaking white water. In addition, the flow velocity of the liquid also impacted the agglomeration of charged particles to a certain extent, making the change more complicated [39]. The results in Table 1 show that the zeta potential, particle size, conductivity, and DS content of the white water after flowing through the PDMS composite pipe increased to different degrees, whereas the turbidity, CD value, pH, and CS content decreased to different degrees. These findings indicate that the white water became more stable after electrical action. After the electrical pretreatment of MOW pulp (Table 2), the negative zeta potential of the pulp increased with the voltage. Electric treatment altered the charge characteristics of the pulp, enhancing the repulsion between the fibers and ink, thereby significantly improving the deinking effect of direct flotation of the pulp. However, chemical deinking was conducted after the electric treatment. This may be because the increased charge inhibited the contact between chemical reagents and the pulp, thus reducing the deinking efficiency.

## 4. Conclusions

In this study, the triboelectric and tensile properties of PDMS were improved by adding PANI-modified cellulose. The tubular triboelectric layer prepared from PDMS exhibited an excellent triboelectric output effect when used in TENG. The results demonstrate the electrical output performance of G-TENG prepared with 5% PANI-modified cellulose and tested with tap water: it achieved a voltage of 22.76 V, a current of 1.24 μA, and a charge of 8.252 nC. In addition, this study demonstrates the successful simulation of the transport state of MOW white water in G-TENG, enabling the collection of its triboelectricity for the pretreatment of MOW pulp. This process enhances the stability of white water and alters the charge characteristics of pulp. The findings highlight the potential of G-TENG as a self-powered sensor power supply and emphasize its application prospect as an energy collection device.

## Figures and Tables

**Table 1:** Changes in properties of MOW white water at different flow rates from G to TENG.

Index	0 mL/min	50 mL/min	200 mL/min	400 mL/min
Zeta potential (mV)	−8.43	−8.69	−8.85	−8.93
Turbidity (NTU)	295	301	275	236
Size (nm)	486	576	603	563
CD (mEq/L)	1.21	1.09	1.12	1.16
Conductivity (μS/cm)	108.1	108.6	110.1	111.8
pH	8.80	8.49	8.56	8.44
DS (%)	0.0319	0.0325	0.0358	0.0338
CS (%)	0.0030	0.0019	0.0011	0.0017

**Table 2:** Properties of MOW pulp before and after G-TENG pretreatment.

Index		0 mL/min	50 mL/min	200 mL/min	400 mL/min
Zeta potential (mV)		−13.1	−13.5	−13.6	−14.3
Direct flotation deinking treatment	Brightness (NTU)	60.30	57.69	59.70	57.92
	ERIC (nm)	134.93	62.62	51.44	64.83
Chemical deinking treatment	Brightness (NTU)	67.35	65.32	69.06	63.99
	ERIC (nm)	23.87	28.54	34.71	50.21

## Data Availability

The data are available in a publicly accessible repository.

## References

[1] Perera F., Nadeau K. (2022). Climate Change, Fossil-Fuel Pollution, and Children’s Health. N. Engl. J. Med..

[2] Wu H. (2022). Trade openness, green finance and natural resources: A literature review. Resour. Policy.

[3] Zhang X., Han L., Wei H., Tan X., Zhou W., Li W., Qian Y. (2022). Linking urbanization and air quality together: A review and a perspective on the future sustainable urban development. J. Clean. Prod..

[4] Puri M., Gandhi K., Kumar M.S. (2023). Emerging environmental contaminants: A global perspective on policies and regulations. J. Environ. Manag..

[5] Wang T., Zhu Q., Zhu Q., Yang Q., Wang S., Luo L. (2022). A highly stable bimetallic organic framework for enhanced electrical performance of cellulose nanofiber-based triboelectric nanogenerators. Nanoscale Adv..

[6] Jiang D., Lian M., Xu M., Sun Q., Xu B.B., Thabet H.K., El-Bahy S.M., Ibrahim M.M., Huang M., Guo Z. (2023). Advances in triboelectric nanogenerator technology—Applications in self-powered sensors, Internet of things, biomedicine, and blue energy. Adv. Compos. Hybrid Mater..

[7] Wang W., Yang D., Yan X., Wang L., Hu H., Wang K. (2023). Triboelectric nanogenerators: The beginning of blue dream. Front. Chem. Sci. Eng..

[8] Mi Y., Lu Y., Shi Y., Zhao Z., Wang X., Meng J., Cao X., Wang N. (2023). Biodegradable Polymers in Triboelectric Nanogenerators. Polymers.

[9] Qi L., Wang J., Dai X., Ning F., Yang P., Chen J., Li Y., Chen J., Zhao Y., Zhang X. (2023). Interspersed Reticulate Cu_2_WS_4_ Nanocrystal–PVDF/Ni Triboelectric Nanogenerators for Rhodamine B Degradation. ACS Appl. Nano Mater..

[10] Wang Z., Liang X., Liu Z., Huang T., Wang S., Yao S., Ding Y., Zhang J., Wan X., Wang Z.L. (2023). Self-powered electrochemical water treatment system for pollutant degradation and bacterial inactivation based on high-efficient Co(OH)_2_/Pt electrocatalyst. Nano Res..

[11] Wolf M.P., Salieb-Beugelaar G.B., Hunziker P. (2018). PDMS with designer functionalities—Properties, modifications strategies, and applications. Prog. Polym. Sci..

[12] Qi D., Zhang K., Tian G., Jiang B., Huang Y. (2021). Stretchable Electronics Based on PDMS Substrates. Adv. Mater..

[13] Miranda I., Souza A., Sousa P., Ribeiro J., Castanheira E.M.S., Lima R., Minas G. (2022). Properties and Applications of PDMS for Biomedical Engineering: A Review. Funct. Biomater..

[14] Sun Z., Yang W., Chen P., Zhang Y., Wang X., Hu Y. (2022). Effects of PDMS Base/Agent Ratios and Texture Sizes on the Electrical Performance of Triboelectric Nanogenerators. Adv. Mater. Interfaces.

[15] Zhu Q., Wang T., Wei Y., Sun X., Zhang S., Wang X., Luo L. (2022). Low-cost, environmentally friendly and high-performance cellulose-based triboelectric nanogenerator for self-powered human motion monitoring. Cellulose.

[16] Sheng Z., Qiuxiao Z., Tingting W., Xuchong W., Xiaoping S., Yuhe W., Lianxin L. (2022). Contact electrification property controlled by amino modification of cellulose fibers. Cellulose.

[17] Bulathsinghala R.L., Ravichandran A., Zhao H., Ding W., Dharmasena R.D.I.G. (2024). The intrinsic impact of dielectric constant on output generation of triboelectric nanogenerators. Nano Energy.

[18] Varghese H., Hakkeem H.M.A., Chauhan K., Thouti E., Pillai S., Chandran A. (2022). A high-performance flexible triboelectric nanogenerator based on cellulose acetate nanofibers and micropatterned PDMS films as mechanical energy harvester and self-powered vibrational sensor. Nano Energy.

[19] Jose D., Jelmy E.J., Subin P.S., Joseph R., John H. (2022). Triboelectric nanogenerator based on polyaniline nanorods incorporated PDMS composites through a facile synthetic route. J. Mater. Sci. Mater. Electron..

[20] Cai Y.-W., Wang G.-G., Mei Y.-C., Zhao D.-Q., Peng J.-J., Sun N., Zhang H.-Y., Han J.-C., Yang Y. (2022). Self-healable, super-stretchable and shape-adaptive triboelectric nanogenerator based on double cross-linked PDMS for electronic skins. Nano Energy.

[21] Wu X.-F., Ge Q.-M., Jiang N., Zhao W.-F., Liu M., Cong H., Zhao J.-L. (2023). Research Progress on Chiral Supramolecular Sensors for Enantiomer Detection. Chemosensors.

[22] Min J., Zhou Z., Fu H. (2023). A self-healing electrostatic interaction crosslinked temperature sensitive conductive hydrogel for strain and temperature sensor. Polym. Adv. Technol..

[23] Rahmani P., Shojaei A., Dickey M.D. (2024). A highly conductive and ultra-stretchable polyaniline/cellulose nanocrystal/polyacrylamide hydrogel with hydrophobic associations for wearable strain sensors. J. Mater. Chem. A.

[24] Lan H., Zhang H., Yang D., Bi S., Liu J., Wang W., Zhang H. (2018). Screening Predominant Bacteria and Construction of Efficient Microflora for Treatment of Papermaking White Water. Bioresources.

[25] Fan Z., Li Z., Qi W., Zhao S., Zhou B., Liu S., Tian Y. (2023). Preparation of in-situ modified diatomite and its application in papermaking. Colloids Surf. A Physicochem. Eng. Asp..

[26] Monte M.C., Blanco A., Negro C., Tijero J. (2004). Development of a methodology to predict sticky deposits due to the destabilisation of dissolved and colloidal material in papermaking—Application to different systems. Chem. Eng. J..

[27] Miranda R., Blanco A., Negro C. (2009). Accumulation of dissolved and colloidal material in papermaking—Application to simulation. Chem. Eng. J..

[28] Ćirić-Marjanović G. (2013). Recent advances in polyaniline research: Polymerization mechanisms, structural aspects, properties and applications. Synth. Met..

[29] Beygisangchin M., Abdul Rashid S., Shafie S., Sadrolhosseini A.R., Lim H.N. (2021). Preparations, Properties, and Applications of Polyaniline and Polyaniline Thin Films—A Review. Polymers.

[30] Yang L., Wang Y., Guo Y., Zhang W., Zhao Z. (2019). Robust Working Mechanism of Water Droplet-Driven Triboelectric Nanogenerator: Triboelectric Output versus Dynamic Motion of Water Droplet. Adv. Mater. Interfaces.

[31] Qin Y., Mo J., Liu Y., Zhang S., Wang J., Fu Q., Wang S., Nie S. (2022). Stretchable Triboelectric Self-Powered Sweat Sensor Fabricated from Self-Healing Nanocellulose Hydrogels. Adv. Funct. Mater..

[32] Zhou M., Wang J., Tan S., Ji G. (2023). Top-down construction strategy toward sustainable cellulose composite paper with tunable electromagnetic interference shielding. Mater. Today Phys..

[33] Chang Z., Liang D., Sun S., Zheng S., Sun K., Wang H., Chen Y., Guo D., Zhao H., Sha L. (2024). Innovative modification of cellulose fibers for paper-based electrode materials using metal-organic coordination polymers. Int. J. Biol. Macromol..

[34] Chang Z., Zheng S., Han S., Qian X., Chen X., Wang H., Liang D., Guo D., Chen Y., Zhao H. (2024). Development of novel paper-based supercapacitor electrode material by combining copper-cellulose fibers with polyaniline. Int. J. Biol. Macromol..

[35] Xu Z., Zhou J., Li D., Zhu G., Lin N. (2023). Flexible Conductive Fibers from Alginate, Cellulose Nanocrystals, and Polyaniline by Wet Spinning. ACS Sustain. Chem. Eng..

[36] Zheng X., Ji B., Jiang R., Cui Y., Xu T., Zhou M., Li Z. (2022). Polydimethylsiloxane/carbonized bacterial cellulose sponge for oil/water separation. Process Saf. Environ. Prot..

[37] Huang X., Ge M., Wang H., Liang H., Meng N., Zhou N. (2022). Functional modification of polydimethylsiloxane nanocomposite with silver nanoparticles-based montmorillonite for antibacterial applications. Colloids Surf. A Physicochem. Eng. Asp..

[38] Van Goethem C., Naik P.V., Van de Velde M., Van Durme J., Verplaetse A., Vankelecom I.F.J. (2023). Stability of Filled PDMS Pervaporation Membranes in Bio-Ethanol Recovery from a Real Fermentation Broth. Membranes.

[39] Gao J., Zhang H., Zhang Q., Li S., Luo B., Sha J., Liu H. (2022). Novel stretchable fiber-shaped fluidic nanogenerators fabricated from carbonized lignin/thermoplastic polyurethane. Ind. Crop. Prod..

